# Discovery of a Protective *Rickettsia prowazekii* Antigen Recognized by CD8^+^ T Cells, RP884, Using an *In Vivo* Screening Platform

**DOI:** 10.1371/journal.pone.0076253

**Published:** 2013-10-16

**Authors:** Michal Gazi, Erika Caro-Gomez, Yenny Goez, Maria A. Cespedes, Marylin Hidalgo, Paula Correa, Gustavo Valbuena

**Affiliations:** 1 Department of Pathology, University of Texas Medical Branch, Galveston, Texas, United States of America; 2 Facultad de Ciencias, Departamento de Microbiología, Pontificia Universidad Javeriana, Bogota, Colombia; 3 Grupo de Inmunomodulación, Facultad de Medicina, Universidad de Antioquia, Medellin, Colombia; 4 Sealy Center for Vaccine Development, Center for Tropical Diseases, Center for Biodefense and Emerging Infectious Diseases, Institute for Translational Sciences, University of Texas Medical Branch, Galveston, Texas, United States of America; Texas A& M Health Science Center, United States of America

## Abstract

*Rickettsia prowazekii* has been tested for biological warfare due to the high mortality that it produces after aerosol transmission of very low numbers of rickettsiae. Epidemic typhus, the infection caused by these obligately intracellular bacteria, continues to be a threat because it is difficult to diagnose due to initial non-specific symptoms and the lack of commercial diagnostic tests that are sensitive and specific during the initial clinical presentation. A vaccine to prevent epidemic typhus would constitute an effective deterrent to the weaponization of *R. prowazekii*; however, an effective and safe vaccine is not currently available. Due to the cytoplasmic niche of *Rickettsia*, CD8^+^ T-cells are critical effectors of immunity; however, the identification of antigens recognized by these cells has not been systematically addressed. To help close this gap, we designed an antigen discovery strategy that uses cell-based vaccination with antigen presenting cells expressing microbe's proteins targeted to the MHC class I presentation pathway. We report the use of this method to discover a protective T-cell rickettsial antigen, RP884, among a test subset of rickettsial proteins.

## Introduction

Epidemic typhus is one of the most lethal infections known to humans; mortality can be as high as 60% without antibiotic treatment [1]. The etiologic agent, *Rickettsia prowazekii*, is an obligately intracellular bacterium transmitted by the human body louse in nature, but it also has the potential for intentional aerosol transmission; indeed, *R. prowazekii* remains on CDC's list of biothreat select agents because of its high mortality, history of development as a bioweapon, transmissibility by aerosol, prolonged infectious stability in louse feces, and ID_50_ of fewer than 10 organisms [2], [3]. Prophylactic vaccines are not currently available to prevent this lethal disease or any of the other rickettsioses. This is a public health priority because clinical diagnosis of rickettsioses is very difficult due to the non-specific initial clinical presentation and the lack of commercially available diagnostic tests that can be used during the acute stage when antibiotic intervention is helpful. To address this need, it will be more cost-effective to produce a cross-reactive vaccine that can also protect against at least the other member of the typhus group *Rickettsia, R. typhi*, the agent of flea-borne murine typhus. This is a prevalent and underdiagnosed infectious disease that is more frequent in rat-infested locations [4]. Although it is clinically milder than epidemic typhus, it causes considerable morbidity.

Until recently, antigen identification for vaccine development was almost exclusively biased towards the humoral immune response. This bias was partly due to the effectiveness of antibodies in protection against almost all of the currently approved vaccines for human use, the relative technical simplicity of working with serum and antibodies, and the methodical challenges of working with T-cells. Presently, the barriers to identify potent vaccine antigens recognized by T-cells need to be addressed because most of the vaccines that remain to be produced require a strong T-cell component to afford significant protection. In particular, there is an urgent need to develop appropriate techniques to identify antigens recognized by T-lymphocytes because antigen discovery is the most important aspect of any vaccine development project; without appropriate antigens, a vaccine is unlikely to succeed.

Given the evidence that CD4^+^ T cells and CD8^+^ T cells target different antigens [5], it is clear that antibody-based screening methods are not suitable to identify antigens recognized by CD4^+^ T cells or, particularly, CD8^+^ T cells. Several approaches to more directly identify antigens recognized by T-cells have been used; many of them rely on Reverse Vaccinology, a branch of Systems Biology that analyses entire microbial genomes to predict immunogenic proteins based on predefined rules derived from the analysis of large empirical datasets [6].

Empirical methods for identification of antigens recognized by T-lymphocytes rely on T-cells from animals or individuals that are immune to the pathogen. Those memory T-cells had been selected during the physiological immune response to persist and recognize a limited number of antigens (i.e., immunodominant antigens). Thus, methods that use memory T-cells for antigen identification are more likely to miss potentially protective subdominant antigens. One strategy for T-cell antigen identification that is not biased towards immunodominant antigens is genomic immunization or Expression Library Immunization (ELI) [7]. Although ELI has been successfully used [8], it has its own problems as it relies on a DNA immunization strategy; thus, antigen expression is not guaranteed in all cases. Accordingly, it is not possible to know which pathogen genes were not screened validly; a negative response can be due to lack of an immunological response or to failed expression of the microbial gene.

Herein, we report the discovery of a conserved typhus group rickettsial antigen using an *in vivo* system of antigen identification that has the potential to address some of the issues raised above because it is not biased by immunodominance, verifies pathogen ORF expression, and can potentially screen a pathogen's entire ORFeome (the collection of all open reading frames from a microbe).

## Materials and Methods

### Cell lines

C1.18.4 (myeloma), I.13.35 (macrophages), LADMAC (transformed bone-marrow cells), and SVEC4-10 (endothelial) cell lines are all derived from C3H mice and were obtained from ATCC. C3HSV cells (fibroblasts) are also derived from C3H mice and were obtained from the Jackson Laboratory. All cell lines were cultivated according to provider instructions. Experiments with SVEC4-10 cells were performed in Advanced DMEM (Gibco) medium supplemented with 3% BGS, 1x Glutamax, and 10 mM Hepes.

### Bacteria

*Rickettsia typhi* (Wilmington strain) is a clinical reference strain with an unknown number of passages in the yolk sacs of embryonated chicken eggs. For all the experiments described in this study, a stock of *R. typhi* was produced in a certified biosafety level 3 (BSL3) laboratory by cultivation in specific pathogen free embryonated chicken eggs. Yolk sacs from infected eggs with dead embryos were homogenized in a Waring blender, diluted to a 10% suspension in sucrose-phosphate-glutamate buffer (SPG; 0.218 M sucrose, 3.8 mM KH_2_PO_4_, 7.2 mM K_2_HPO_4_, 4.9 mM monosodium L-glutamic acid, pH 7.0) and aliquoted for storage at −80°C after discarding the pellet produced by low speed centrifugation (200 × g, 10 minutes). Rickettsial content of this stock was quantified by plaque assay [9], and the LD_50_ was determined experimentally in C3H/HeN mice.

### Animal model and ethics statement

The mouse model of endothelial-target typhus group rickettsioses consists of *Rickettsia typhi* infection of C3H/HeN mice (Charles River Laboratories, stock 025) and has been previously described in detail [10]. All mice were housed in an animal biosafety level-3 (ABSL3) facility and were infected intravenously (through the tail vein) with 3 LD_50_ of *R. typhi* in a volume of 300 μl of phosphate-buffered saline (PBS).

We followed the recommendations in the Guide for the Care and Use of Laboratory Animals of the National Institutes of Health. For survival analyses, we used a clinical scoring system to replace death as an endpoint; once animals became anorexic and inactive with a rough coat, they were observed at least twice daily. Animals that showed immobility with lack of response to external stimuli and dehydration were euthanized with CO_2_ following current AVMA guidelines. Analgesics were not used due to their known effects on inflammatory pathways that can affect outcomes after vaccination and challenge. Our experimental protocol was approved by the Institutional Animal Care and Use Committee (IACUC) of the University of Texas Medical Branch (protocol number: 0903026).

### Eukaryotic expression vector

pDEST-M1, our eukaryotic expression vector, was assembled in four cloning steps. The ptdTomato-N1 plasmid (Clontech Laboratories) was used as a backbone. The Destabilization Domain (DD) from the pDD-tdTomato plasmid (Clontech Laboratories) was PCR amplified using primers DD-NheI-forward (5′- AGCTAGCATGGGAGTGCAGGTGGAAACC -3′) and DD-BglII-reverse (5′- AGAGATCTCTTTCCGGTTTTAGAAGCTCC-3′), and cloned into ptdTomato-N1 linearized with NheI and BglII. The second component, Ubiquitous Chromatin Opening Element (UCOE) was a generous gift from Dr. Michael Antoniou. To add Ase I restriction sites, UCOE was PCR amplified using primers UCOE-AseI-forward (5′-AATTAATCTACAGCTCAAGCCACATCCGA-3′) and UCOE-AseI-reverse (5′- AATTAATGAGACGCCGTGGCCCCCGAAGC-3′). The AseI site was used to add UCOE to our expression vector upstream of the CMV promoter. The third component was the Ampicillin resistance gene, which replaced the Kanamycin-Neomycin resistance gene originally present in the ptdTomato-N1 backbone. The ampicillin resistance gene was removed from pcDNA-DEST47 plasmid (Invitrogen) using BspHI and cloned into our expression vector already containing DD and UCOE; linearization of this vector with BspHI also removed the original Kanamycin-Neomycin resistance gene. The fourth step was to insert the Gateway system (Invitrogen) cassette, which was removed from the pcDNA-DEST47 plasmid (Invitrogen) with HindIII and cloned into our expression vector linearized with the same enzyme. All manipulated parts of the vector were sequenced.

We received 746 *Rickettsia prowazekii* entry clones produced by PCR (in the pDONR 221 vector) from the J. Craig Venter Institute (JCVI). Those genes that could not be cloned by JCVI were made synthetically by GeneArt with codon optimization for eukaryotic expression. We used the Clonase LR II Enzyme Mix (Life Technologies) to transfer individual rickettsial genes from the pDONR221 vector into our eukaryotic expression vector following the manufacturer's recommendation. Positive clones were verified by sequencing and transfection-grade plasmid DNA was isolated with the QIAGEN EndoFree Plasmid Maxi Kit.

### Nucleofection of APCs and vaccination procedures

We used the Amaxa SE Cell Line 96-well Nuclefector kit (Lonza) to nucleofect expression vectors carrying *R. prowazekii* genes into SVEC 4–10 cells expressing CD137L and CD80. For each nucleofection reaction, 4×10^5^ cells were resuspended in 20 μl of SE Nucleofection solution and mixed with 1 μg of plasmid DNA. Cells were nucleofected in the Amaxa 96-well Shuttle Nucleofector device using the program DS-104 SE. Cells were then placed into 6-well plates to recover overnight. The following morning, we checked for the expression of the tdTomato fluorescent protein in our APCs using an inverted fluorescence microscope (Olympus IS71) equipped with a TRITC filter.

Approximately 4×10^5^ cells from each well of a 6-well plate were detached with Accumax (Millipor) and washed with PBS. For the initial screening, five wells expressing different *R. prowazekii* proteins were pooled together (see table S1) and resuspended in 1.5 ml of PBS. That cell suspension was used to immunize 5 mice via the intravenous (i.v.) route, where each mouse received 300 µl of cell suspension (4×10^5^ cells). Four days later, mice received the same dose of cells but this time intraperitoneally (i.p.). Ten days after the second immunization, mice were infected intravenously with 3 to 6 LD_50_ (depending on the experiment) of *R. typhi*. On day 7 post-infection, mice were euthanized, and liver, lung, and spleen were collected for DNA isolation and bacterial load determination. Approximately 10 mg from each collected organ were placed into 2-ml microcentrifuge tubes (Eppendorf) together with one stainless steel grinding ball (5/32′′, Fisher Scientific) and 50 µl of PBS. Samples were homogenized in a TissueLyser II (QIAGEN) operating at 30,000 Hz for 2 minutes. After centrifugation (1 min. at 13,000 rpm), the homogenate was processed for DNA isolation using QIAGEN's DNeasy Blood & Tissue Kit. For deconvolution experiments, all wells of a 6-well plate expressed the same rickettsial protein; the rest of the procedures were the same.

### Statistics

The proportion of surviving animals was analyzed with the Log-rank (Mantel-Cox) test. Mean absolute counts of CD8^+^ T-cell subpopulations were compared using unpaired t-tests.

Additional methods are presented in the Supplementary Methods section.

## Results

### Design of the system

**Figure 1:**
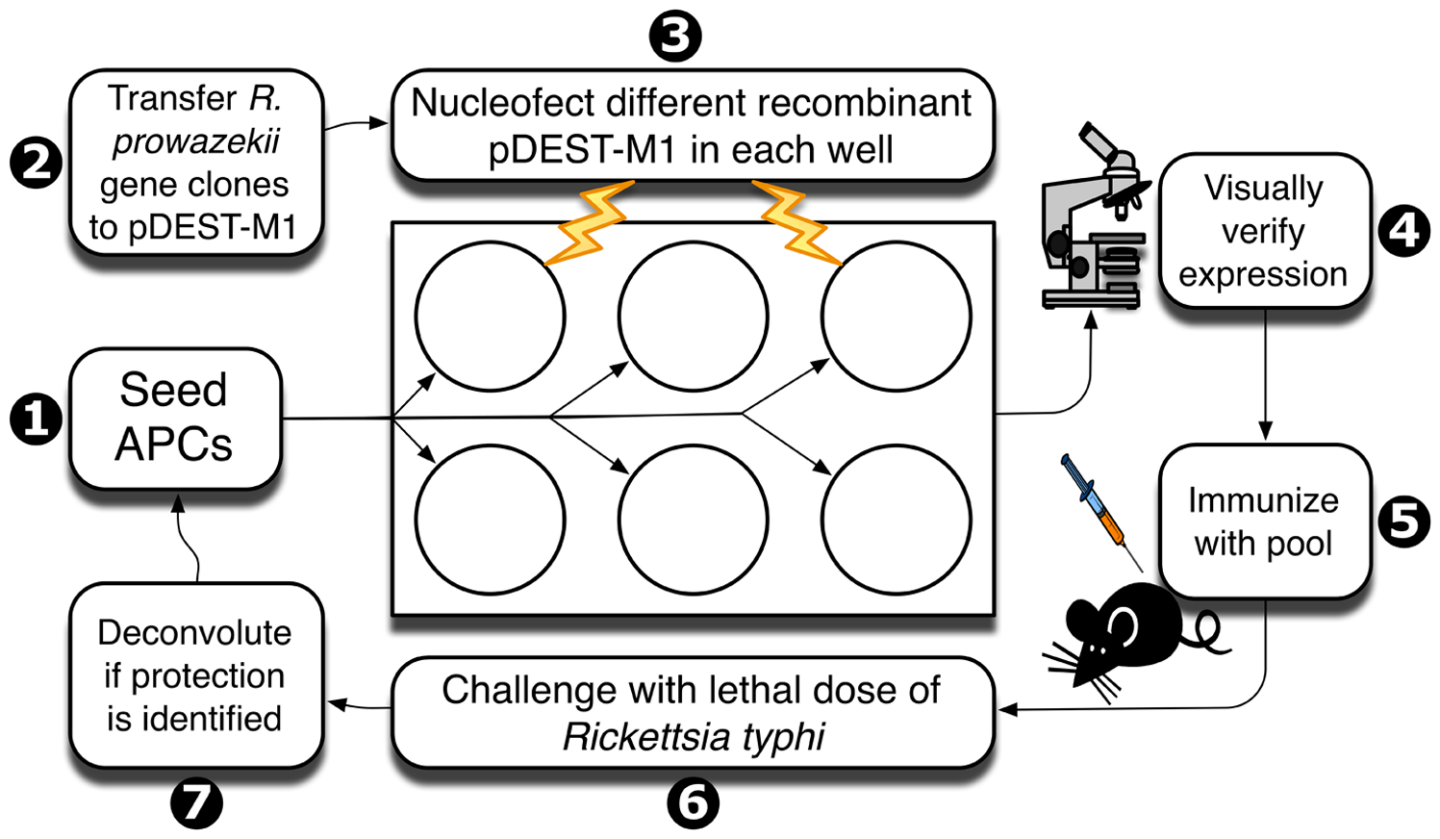
Diagram of the methodology to discover antigens recognized by T-cells. The steps involved are numbered as follows: 1) selection of appropriate antigen presenting cells (APCs) based on MHC class I expression, 2) transfer of *R. prowazekii* gene clones to an eukaryotic expression vector, 3) expression vector transfer to APCs, 4) visual screening to verify expression of rickettsial gene, 5) APC-based immunization of naive mice using pools of 4–6 different APCs expressing rickettsial genes, 6) challenge with *R. typhi* and determination of bacterial load as indicator of protection, and 7) identification of protective rickettsial genes by deconvolution of protective pools.

The overall concept of our vaccine discovery strategy is presented in figure 1; it involves the following steps: 1) selection of appropriate antigen presenting cells (APCs), 2) transfer of *R. prowazekii* gene clones to an eukaryotic expression vector, 3) nucleofection of APCs, 4) visual screening to verify expression of rickettsial gene, 5) APC-based immunization of naive mice using pools of 4–5 APCs expressing different rickettsial genes, 6) measurement of rickettsial load after lethal challenge with *R. typhi*, and 7) identification of protective rickettsial genes by deconvolution of protected pools. Note that this system allows for the detection of cross-reactive responses since the rickettsial genes are derived from *R. prowazekii* but the challenge is performed with *R. typhi*, the other member of the typhus group *Rickettsia*.

### Identification and improvement of antigen presenting cells

We first identified appropriate antigen presenting cells (APCs) by screening several cell lines derived from C3H mice (C1.18.4, I.13.35, LADMAC, SVEC4-10, and C3HSV) for their expression of MHC class I (H2K^k^); they had to be from this mouse strain because C3H mice infected with *R. typhi* faithfully model human typhus group rickettsioses [10]. The SVEC4-10 cell line, an endothelial cell line, had the highest levels of expression of MHC class I among the 5 different cell lines tested (data not shown). We felt confident that this cell line was appropriate for further development because rickettsial antigen presentation by this cell line has been demonstrated in prior studies [11], [12]. In order to further improve SVEC4-10 cells as APCs, we transduced them to express the co-stimulatory molecules CD80 and CD137L using lentiviruses expressing cloned cDNA of mouse CD80 and CD137L (figure S1). *Rickettsia*-infected modified SVEC4-10 cells expressing CD80 and CD137L were better stimulators of IFN-γ production from immune splenocytes than unmodified SVEC4-10 cells (data not shown).

### Production of eukaryotic expression vector and expression of rickettsial genes

**Figure 2:**
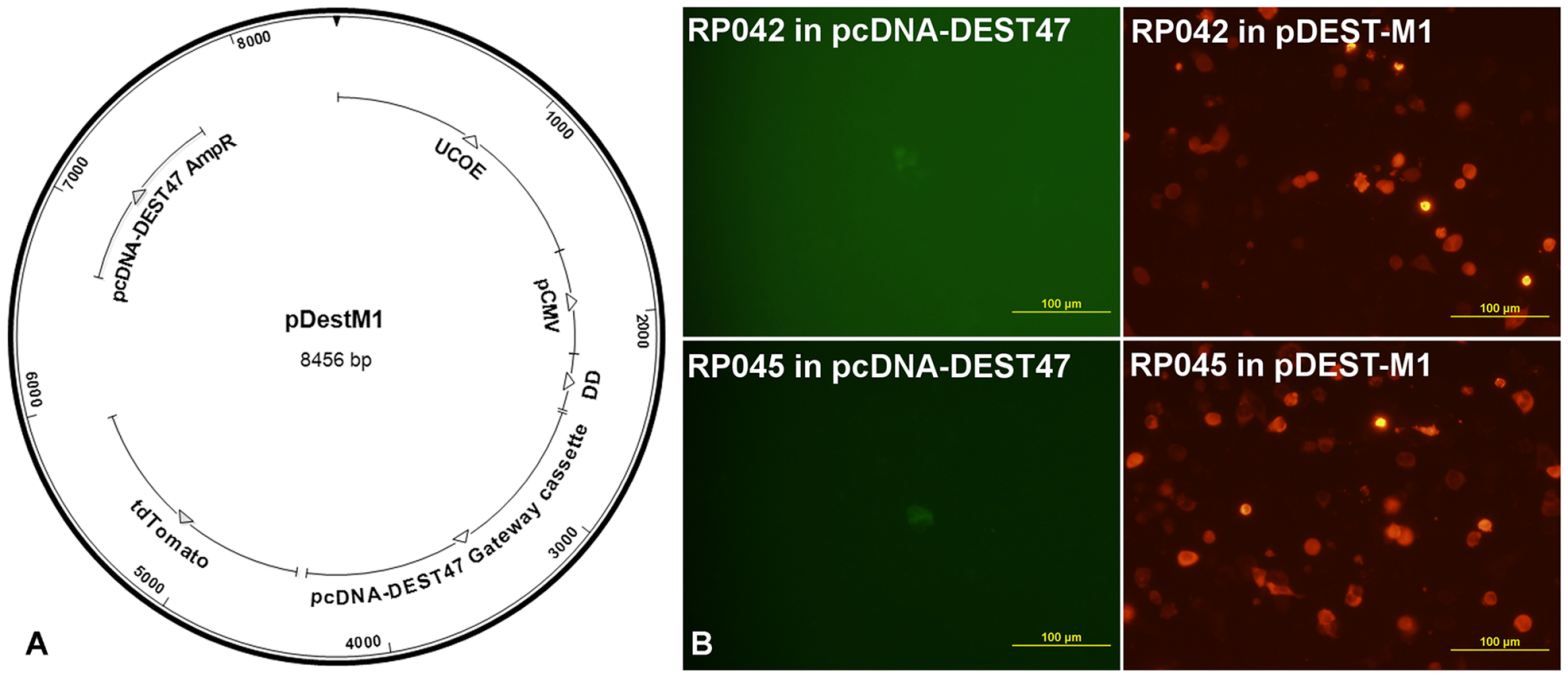
Characteristics of new eukaryotic expression vector. A) Diagram of plasmid components. B) Comparison of the same rickettsial proteins expressed as fusion proteins with GFP (pDEST47) or with our new vector, pDEST-M1. Note the visual difference between signal and background.

We have the entire collection of open reading frames (ORFs) from *R. prowazekii* in the vector pcDNA-DONR 221); 89% of them were provided by the J. Craig Venter Institute while the rest were synthesized by a commercial vendor with codon optimization for mammalian expression; all rickettsial gene clones are in entry plasmid vectors for the Gateway system (Life Technologies) to facilitate transfer to other vectors through site-specific recombination [13]. We first transferred rickettsial genes to the Gateway destination vector pcDNA-DEST47, a commercial eukaryotic expression vector that produces a carboxy-terminus fusion protein with GFP. The purpose was to verify the expression of the rickettsial protein by wide-field fluorescence microscopy after nucleofection of APCs. Since it was difficult to clearly discern the expression of the rickettsial genes based on GFP-expression with this vector, we designed and produced a new Gateway destination vector (pDEST-M1) that could satisfy the requirement for visual screening of fusion protein expression (figure 2A). The new vector allows the expression of rickettsial genes as fusion proteins with Tomato (Clontech), a very bright fluorescent protein, under a strong CMV promoter preceded by a Ubiquitously acting Chromatin Opening Element (UCOE) that leads to stronger and stable expression [14]. This plasmid vector also directs the expressed protein towards presentation through the MHC class I pathway because it has a sequence corresponding to a Destabilization Domain (DD) that is expressed on the N-terminus of the cloned rickettsial gene; its activity leads to rapid proteasomal degradation [15]. The advantage of this system is that expression can still be verified by the addition of a stabilizing agent (Shield, Clontech).

**Figure 3:**
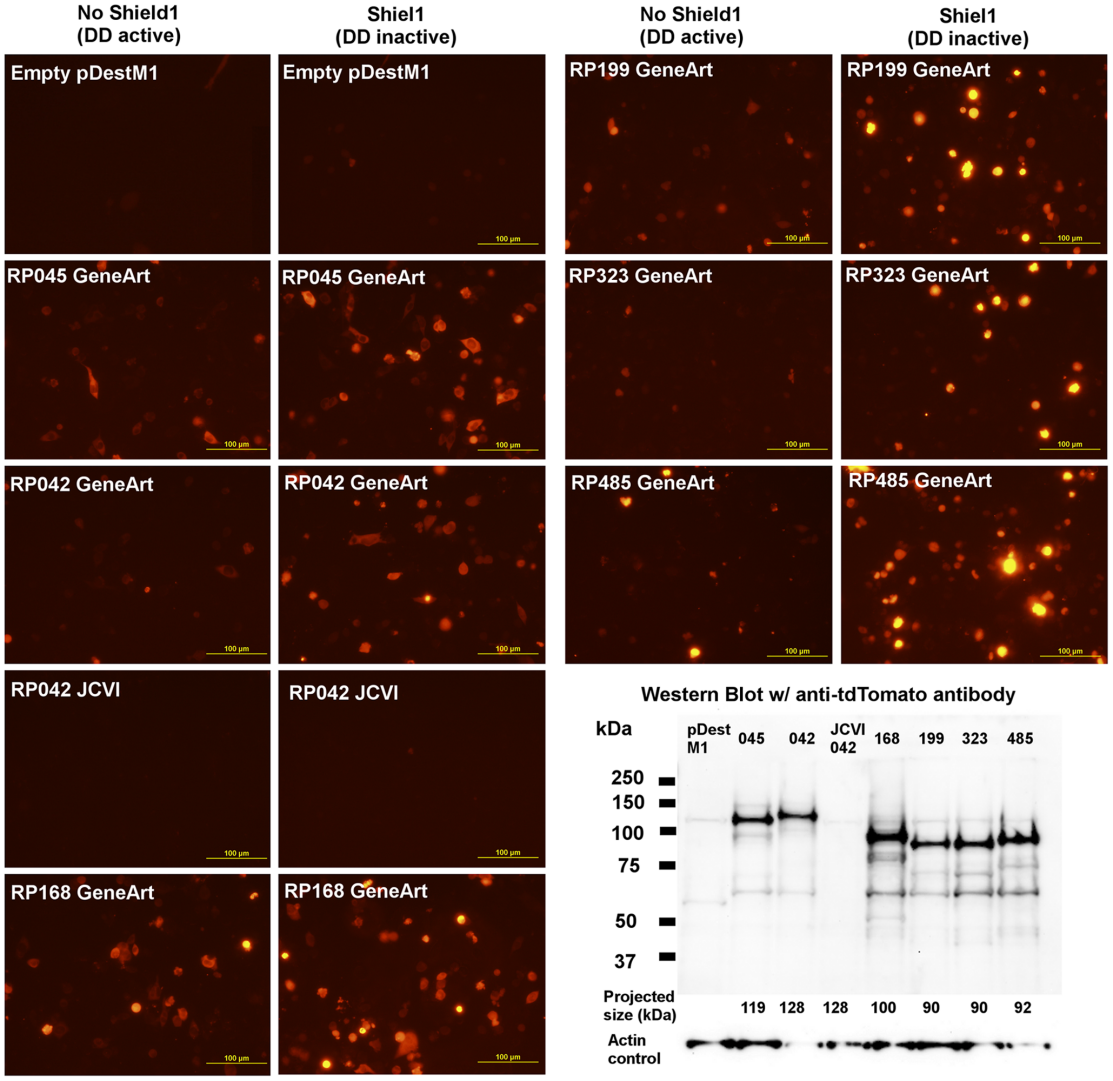
Verification of expression of rickettsial proteins in APCs. Examples of *R. prowazekii* proteins expressed by SVEC4-10 cells as detected by fluorescence microscopy (with and without Shield, a blocker of the destabilization domain) and western blot. Note that RP042 is expressed when codons are optimized for eukaryotic expression (GeneArt) but not when using native rickettsial codons (JCVI).

Previous work with *Rickettsia* has focused on surface proteins for the discovery of epitopes recognized by CD8^+^ T-cells [5], [12], [16], [17]. However, there is evidence that the antigens recognized by antibodies (e.g., surface antigens), which are similar to the ones recognized by CD4^+^ T cells, tend to be different from the ones recognized by CD8+ T cell responses. Also, any protein encoded by a microbial genome, independently of its physical location in the microbial cell, can theoretically be processed for presentation through the class I pathway. Thus, we considered valid to randomly select and test 36 rickettsial genes in our collection. The expression of the selected genes was clearly brighter with our new vector (figure 2B). We also verified that they can be detected in the presence of Shield and that they are degraded in its absence (figure 3). Moreover, we verified the expression of several rickettsial genes as fusion proteins by western blot analysis (figure 3). Interestingly, in one case in which we had the rickettsial gene with both native codon usage as well as optimized for mammalian codon usage (RP042 in figure 3), we found that only the codon optimized version was detectable by fluorescence microscopy or western blot.

### Testing of the system for CD8^+^ T-cell antigen identification

**Figure 4:**
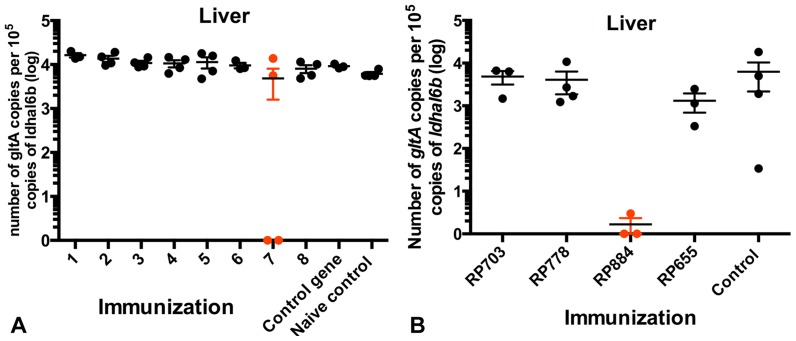
*In vivo* identification of rickettsial antigens. A) Thirty-six APC lines expressing high levels of individual *R. prowazekii* proteins were randomly combined into 8 pools. Eight groups of mice (4 mice per group) were immunized with pools of APCs expressing 4 or 5 different *R. prowazekii* proteins (see table S1) by inoculating 2×10^5^ APCs i.v. As a control, one group of mice was immunized with APCs expressing a gene from *A. thaliana*. We also included a group without any manipulation (blank control). Fifteen days after immunization, all mice were challenged with 3×LD_50_ *R. typhi*. At day 7 post-infection, all animals were terminated, and rickettsial load in liver and lungs (not shown) was determined using quantitative real-time PCR targeting the rickettsial gene *gltA* and the mouse gene *ldhal6b*. We identified one group of mice with a significantly lower load of *Rickettsia*. B) This group was deconvoluted by immunizing mice with the individual constructs following a similar strategy as the one above. We found one protective rickettsial protein in this group, RP884.

We produced APCs expressing high levels of rickettsial proteins encoded by the 36 randomly selected rickettsial gene clones and combined them into 8 random pools (see table S1). Eight groups of C3H/HeN (H2-K^k^) mice were immunized with the pools of MHC-haploidentical APCs expressing *R. prowazekii* proteins (4 or 5 rickettsial proteins per pool) by inoculating 4×10^5^ APCs i.v. and the same amount i.p. four days later. Control groups included one group that was immunized with APCs expressing a control gene from *A. thaliana* and one group that did not receive any immunization treatment (naive control). Fourteen days after initial immunization, all mice were challenged with 4×10^4^ pfu of *R. typhi* (approximately 3× LD_50_). On day 7 post-infection, all animals were terminated, and rickettsial load in liver and lungs, two critical target organs, was determined using quantitative real-time PCR. We identified one group of mice with a significantly lower load of *Rickettsia* (figure 4A). To identify the individual rickettsial component(s) that were mediating protection, we immunized separate groups of naive mice with nucleofected APCs expressing each individual rickettsial gene from the positive pool (RP884, RP703, RP778, RP655) and repeated the challenge procedure as above. The new bacterial load analysis showed that RP884 was the rickettsial gene triggering the protective immune response of the original pool as assessed by a significant decrease in rickettsial load (figure 4B). RP884 is annotated in the *R. prowazekii* genome as a ferrochelatase (*hemH*), an enzyme that participates in protoheme biosynthesis. This protein is highly conserved among the Rickettsiaceae and it is 95% identical to that of *R. typhi*. Outside the Rickettsiales, the maximum protein sequence identity using BLASTp was 44%.

### Efficacy of vaccine antigen candidate and mechanism of protection

**Figure 5:**
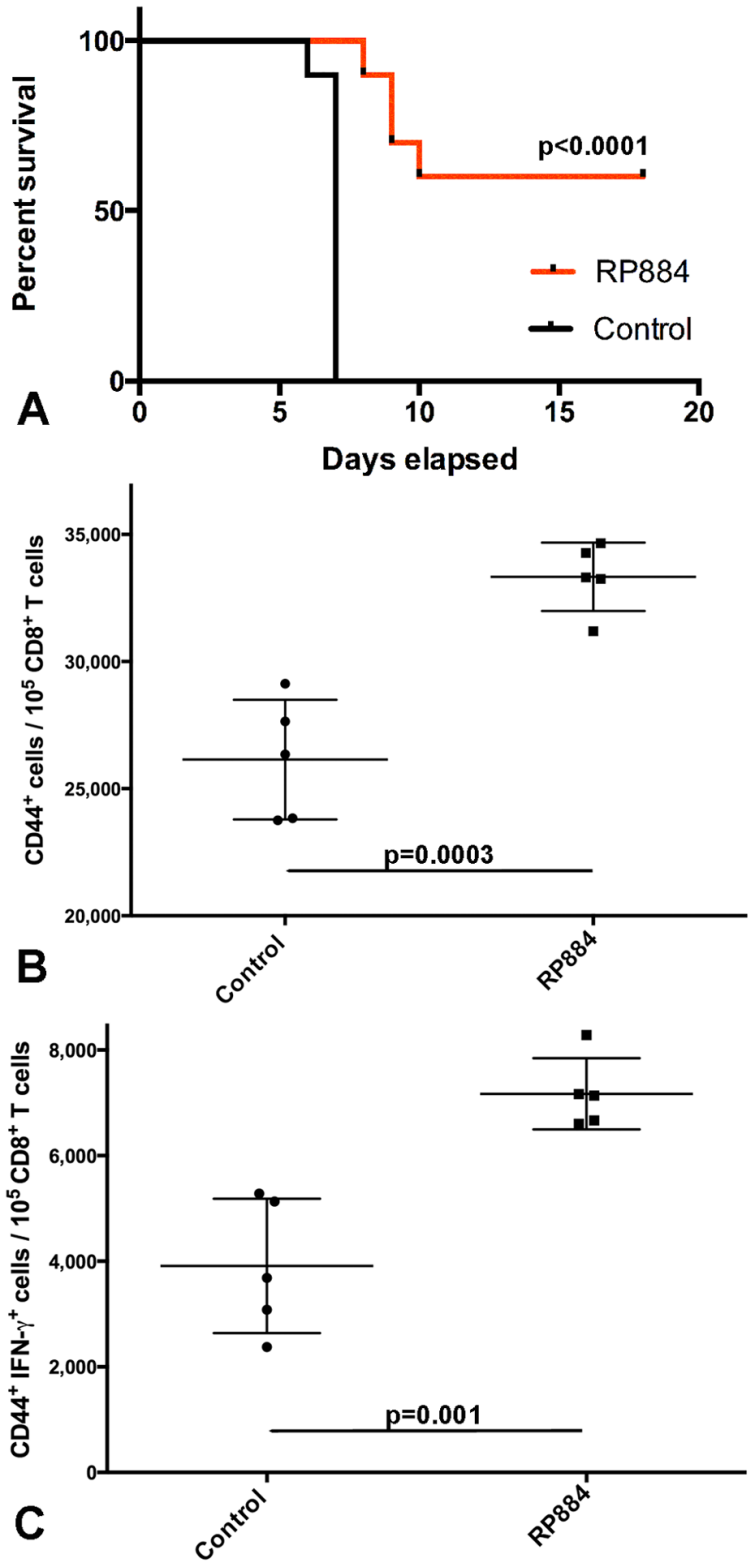
Efficacy of RP884 as a protective antigen and mechanism of protection. A) Naive mice immunized with 2×10^5^ APCs expressing either RP884 or an *A. thaliana* gene (control) were challenged with 3×LD_50_ of *R. typhi* and followed in time to determine survival; 60% of RP884-immune animals survived while none of the control animals did (p<0.0001). B and C) RP884-immune animals and mice immunized with the *A. thaliana* control gene were challenged with 6× LD_50_ of *R. typhi* and sacrificed 7 days later (4 hours after i.p. injection of brefeldin A and monensin) to obtain splenocytes for flow cytometric analysis; cells were stained with antibodies against CD3, CD8, CD44, IFN-γ, and granzyme B to determine the proportion of antigen-experienced CD8^+^ T-cells (B) stimulated to produce IFN-γ (C) *ex vivo* after rickettsial challenge. Differences were statistically significant (p = 0.0003 for panel B and p = 0.001 for panel C). We show individual data points, mean, and standard error of the mean (SEM).

We immunized mice with 4×10^5^ APCs expressing RP884 and challenged them with 3× LD_50_ of *R. typhi*. As shown in figure 5A, 60% of the immunized animals survived while 100% of control animals succumbed to the rickettsial infection. There are at least two possible ways by which our immunization strategy results in T-cell priming. One is direct priming mediated by our modified APCs; the other is cross-presentation by endogenous APCs. Although the aim of our study did not include determining with certainty the pathway(s) that were operational, we did test the survival of SVEC4-10 cells inoculated in C3H/HeN mice to ascertain whether it was at least possible for these cells to function in direct antigen presentation *in vivo*. This cell line was transduced to express a luciferase gene (*Luc2*) in order to be able to track them through repetitive imaging (figure S2). The signal rapidly declined after intravenous inoculation; however, it persisted up to 6 days after i.p. inoculation. Since the i.v. injection method produces rapid dilution and re-distribution of the inoculum, we believe that the signal is not detectable because it is not concentrated in a single space as is the case for the i.p. innoculation. To validate this notion, we also injected *Luc2*-expressing cells i.m. in order to detect a concentrated signal in a single anatomical space; the signal was also detectable at a stable level for 6 days and then disappeared. This result indicates that SVEC4-10 cells expressing a foreign antigen can persist up to the time when effector T-cells would be expected to be circulating and active. Therefore, this result does not rule-out direct antigen presentation by our synthetic APCs.

The immunization strategy for antigen discovery was designed to be biased towards MHC class I presentation because the antigens are rapidly delivered to the proteasome as a consequence of the fusion of the rickettsial protein to a destabilization domain. To explore the role of class I presentation, we analyzed the effector functions in T-cell subpopulations from immunized animals. We used an *ex vivo* method that provides direct information about the response of T-cells *in vivo* because T-cells are stained and analyzed immediately after their extraction without further *in vitro* stimulation [18], [19]. Mice were injected with brefeldin A and monensin, two substances that stop secretory processes, four hours before retrieval of the spleen in order to detect cytokines that were being secreted by T-cells at the time of the injection. *Ex vivo* analysis of CD3^+^ CD8^+^ cells from animals immunized with SVEC4-10 cells expressing RP884 showed increased numbers of CD44^+^ cells among CD8^+^ T-cells. Since CD44 is a surface marker expressed by antigen-experienced T-cells, its increased expression suggested that RP884 immunization efficiently primed CD8^+^ T cells (CD3^+^ CD8^+^ CD44^+^ cells, figure 5B). Furthermore, this subset also produced increased levels of IFN-γ after rickettsial challenge compared to control animals immunized with SVEC4-10 cells expressing an irrelevant antigen (control gene, figure 5B); increased production of granzyme B was also observed in RP884 immune animals, although it was not statically significant at the traditional level (p = 0.058). Effector CD8^+^ T-cells (CD44^+^ CD127^lo^) and memory CD8^+^ T-cells (CD44^+^ CD127^hi^) were also increased in RP884-immune animals (figure S3), and significantly larger numbers of IFN-γ-producing cells were observed among these subpopulations (figure S4). On the other hand, responses of antigen-experienced CD4+ T-cells (CD4^+^ CD44^+^) were not significantly different (data not shown). These data indicate the development of an anti-RP884 CD8^+^ T-cell response in immunized animals, and supports the concept that our antigen discovery strategy functions through stimulation of CD8^+^ T-cell responses as expected.

### Predictive power of immunoinformatic tools

Since our strategy for antigen discovery is entirely empirical, we wanted to compare our results to those generated with publicly available bioinformatic systems that predict the immunogenicity of proteins toward CD8^+^ T-cells. The algorithms supporting each system are different; thus, each one produces a different ranking. We used the individual scores generated by each server and also ranked *R. prowazekii* proteins using two different scoring systems: a) Single peptide score (SpS) and b) overall protein score (OpS). For SpS, rickettsial proteins were ranked according to the inverse IC_50_ value of the predicted peptide with highest affinity in each protein so that the rickettsial protein containing the peptide with the lowest IC_50_ value was ranked first; we used only NetMHCpan and IEBD-ANN since both servers can generate IC_50_ values. The SpS score resulted from averaging the rank generated with each one of the two servers. This score included proteins with high-affinity MHC class-I-binding peptides regardless of the number of potential predicted peptide binders in each protein.

The purpose of the OpS score was to weight rickettsial proteins according to the number of MHC class I binding peptides that can be potentially generated from each one. We first generated a protein score (pS) for each server from the inverse of the average of the IC_50_ values (for NetMHCpan or IEBD-ANN) and the S-Score (for SYFPEITHI) of all peptides predicted to bind to H-2K^k^ from each rickettsial protein divided by the number of predicted peptides binders in the same protein. The OpS score was calculated from the average of the three pS ranks.

As shown in table 1, RP884 ranked rather low with all systems used, suggesting that individual scores from these servers or our calculated SpS and OpS scores were not optimal predictors. To overcome this problem, we redefined our analysis strategy as follows: First, we used NetMHCpan, IEBD-ANN, and SYFPEITHI to generate a database of high-affinity MHC class I binding peptides for each protein. Then, we used RANKPEP to query the top 5 proteasome-derived peptides among all the MHC class I binding peptides predicted for each protein. With this information, we generated a manually curated database that included only proteasome-derived peptides with their respective RANKPEP scores (calculated by dividing the individual peptide scores by the optimal score value). With this information, a new rickettsial protein score was obtained by multiplying the number of proteasome predicted peptides found among all the predicted MHC class I-binders in each protein by the average of the RANKPEP scores for each protein. After performing these modifications to our *in silico* analysis strategy, RP884 emerged as a top-ranked *in silico*-defined vaccine candidate (New modified score, Table 1). These results highlight the importance of combining proteasome-processing as well as MHC class I binding predictions for *in silico* approaches aimed at discovering CD8^+^ T-cell antigens.

**Table 1:** Immunoinformatic analysis and immunogenicity ranking of *R. prowazekii* proteins.

	Rank of positive pool genes among all *R. prowazekii* proteins (834)				Rank of positive pool genes among those tested in this study (36)			
Predictors	RP655	RP703	RP778	RP884	RP655	RP703	RP778	RP884
NetMHCpan	790	185	11	650	34	16	2	32
IEBD-ANN	795	135	35	197	35	16	6	18
SYPEITHI	624	308	15	311	33	26	2	24
SpS score	807	48	173	358	35	3	16	18
OpS score	781	169	10	392	35	20	1	26
New modified score	518	129	38	4	35	10	8	3

## Discussion

*Rickettsia prowazekii*, the intracellular bacterium that causes epidemic typhus, is among the most lethal pathogens affecting mankind. It has, in fact, changed the course of history by decimating armies and civilian populations during times of war [20], [21] and it has been used as a biological weapon [3]. Also, epidemic typhus is the only known rickettsiosis that can present in a recrudescent form years after the primary infection, a condition known as Brill-Zinsser disease [22]. This form of epidemic typhus is a source of new epidemics if it occurs at a place and time with deteriorated living and hygiene conditions that would favor the presence of the human body louse, its vector.

Despite the many efforts to develop a vaccine against *R. prowazekii* [23], no vaccine is currently available; the production of the most recent vaccine was stopped due to its significant variability in antigenicity and potency [24]. More recent work was aimed at the development of a subunit vaccine using the rickettsial surface proteins OmpA [25], [26] and OmpB [12], [27] as potential targets for CD8^+^ T-cells. The feasibility of a subunit vaccine that triggers T-cell-mediated cross-protection against typhus group *Rickettsia* is supported by the following: 1) T-cell cross-reactivity is favored by the T-cells themselves since the T-cell receptor is multispecific due to structural flexibility and interactions with only a few residues within the presented peptides [28], [29]; 2) T-cell-mediated cross protection has been demonstrated between organisms as genetically distant as *Aspergillus* and *Candida* [30] or as diverse as influenza virus [31], 3) T-cells are critical for effective immune responses against all *Rickettsia*, which is congruent with their intracellular cytoplasmic niche [32], [33]; 4) the anti-rickettsial T-cell response is sufficient for protection as demonstrated through T-cell transfer studies [32], [33]; and 5) these T-cell responses are naturally cross-protective within the two major rickettsial groups (typhus and spotted fever groups) [34] and, as demonstrated by us, even between those major groups [11].

In this article, we report the discovery of an antigen recognized by CD8^+^ T-cells that is cross protective between the two members of the typhus group *Rickettsia*, *R. prowazekii* and *R. typhi*. The discovered antigen from *R. prowazekii*, RP884 (annotated as a ferrochelatase), is highly conserved among members of the family Rickettsiaceae and it is 95% identical to that of *R. typhi*, which is consistent with our finding of cross protection. Cross reactivity outside the order Rickettsiales would not be expected given the low sequence conservation outside this order. This is a step forward toward the development of a subunit vaccine against typhus group *Rickettsia* by providing a relevant protective antigen that can be combined with other antigens to be identified in the future. We used a novel antigen discovery platform as illustrated in figure 1. The idea is to easily produce APCs expressing individual ORFs from a sequenced pathogen and use them for immunization of naive mice. Immunization with pooled APCs containing 4 to 5 pathogen's ORFs is followed by challenge with live virulent pathogen and measurement of an indicator of protection such as decreased bacterial load. Once protective pools are identified, each member of the pool is tested individually (deconvolution) to identify ORF(s) responsible for a protective immune response. With this platform, one can test for cross-protective responses by immunizing with the ORFs of one pathogen (*R. prowazekii* in this case) and challenging with a related microbe (*R. typhi*). Importantly, the proposed methodology is not biased by immunodominance because T-cells from immune animals are not used to select antigens. This aspect is potentially important for vaccine development because subdominant or cryptic antigens have been shown to elicit protective immune responses in other systems [35]-[37]. It is interesting to note that the initial positive pool containing RP884 and other 3 antigens showed evidence of protection in only half of the animals. One possible explanation that we intend to test in the future is that one or more of the other antigens in the pool trigger a suppressive response.

The platform described here has the potential to discover relevant antigens for vaccine development independently of their ranking in the natural hierarchy of immunodominance, which dramatically expands the universe of possible antigens; thus, it offers a possible solution to the identification of protective antigens that are conserved among different strains of a microbe or even different species within a genus. This platform also has the capability of identifying multiple antigens from a pathogen, which can then be used as components of a subunit vaccine. Indeed, our results demonstrate that a single antigen from *R. prowazekii*, the product of the RP884 gene, can stimulate CD8^+^ T-cells and immunize mice against a challenge with *R. typhi* (figures 4 and 5). The resulting immune effector and memory CD8^+^ T-cells (figures S3 and S4) produced IFN-γ, a known critical effector of the anti-rickettsial immune response [10], [38], [39].

Our findings are also in agreement with the concept that codon-optimization may be very important not only for DNA vaccine design [40] but also for screening strategies for antigen discovery (figure 3). Moreover, the fact that the protective antigen reported here was not ranked highly by several different immunoinformatic programs (table 1) suggests that there is a need for further development on that front and that empirical approaches are feasible and relevant. This is not necessarily surprising since the predicting power of those immunoinformatic strategies has not been directly compared against an empirical approach testing all ORFs (the ORFeome) of a pathogen. Moreover, at least for bacterial proteins, known protective antigens actually have less predicted epitopes than randomly selected bacterial protein sets used as a control [41].

Future experiments with MHC class I-deficient mice (and adoptively transferred naive CD8^+^ T-cells) will directly address if direct antigen presentation is the mechanism of T-cell priming of our antigen-screening system. We will also continue to scan the *R. prowazekii* ORFeome with the same novel platform described here to broaden the validation of our antigen screening strategy and to discover new antigens that may be introduced in a future safe and effective cross-reactive subunit vaccine against both typhus group *Rickettsia*, *R. typhi* and *R. prowazekii*.

## Supporting Information

Figure S1 **Selection and modification of antigen presenting cells (APCs).** SVEC4-10 cells transduced with lentiviruses to express CD137L and CD80 were selected with puromycin and subsequently sorted by fluorescence-activated cell sorting (FACS) to isolate only cells expressing high levels of these proteins. Selected cells were expanded and analyzed by flow cytometry before nucleofection. A representative example of expanded transduced SVEC4-10 cells is shown.
(TIF)

Figure S2 *In vivo* imaging of antigen presenting cells (SVEC4-10 cells) expressing luciferase at the indicated times after intraperitoneal or intramuscular injection. A) Average radiance. B) Luminescence of luciferase-expressing cells in the presence of luciferin (upper left well) vs. control cells (other wells). C) Luminescent signal from a mouse inoculated intravenously with 4×10^5^ cells expressing luciferase one hour after inoculation (average radiance is not shown in A because no luminescence was observed after one day). D) Luminescent signal from a mouse inoculated intramuscularly (i.m.) two days earlier with 4×10^5^ cells expressing luciferase. E) Luminescent signal from a mouse inoculated intraperitoneally (i.p.) two days earlier with 4 x 10^5^ cells expressing luciferase.
(TIF)

Figure S3 **Increased effector and memory CD8+ T-cells in mice immunized with RP884.** RP884-immune animals and mice immunized with the *A. thaliana* control gene were challenged with 6× LD_50_ of *R. typhi* and sacrificed 7 days later to obtain splenocytes for flow cytometric analysis; cells were stained with antibodies against CD3, CD8, CD44, and CD127 to determine the proportion of antigen-experienced CD8^+^ T-cells with an effector (panel A) or memory (panel B) phenotype based on the expression of CD127. We show individual data points, mean, and standard error of the mean (SEM).
(TIF)

Figure S4 **Increased IFN-γ-producing effector and memory CD8+ T-cells in mice immunized with RP884.** RP884-immune animals and mice immunized with the *A. thaliana* control gene were challenged with 6× LD_50_ of *R. typhi* and sacrificed 7 days later (4 hours after i.p. injection of brefeldin A and monensin) to obtain splenocytes for flow cytometric analysis; cells were stained with antibodies against CD3, CD8, CD44, CD127, and IFN-γ to determine the proportion of antigen-experienced CD8^+^ T-cells that produce IFN-γ among effector (panel A) and memory (panel B) cells. We show individual data points, mean, and standard error of the mean (SEM).
(TIF)

File S1 **Supplementary Materials and Methods.**
(DOCX)

Table S1 **Pool assignment of** ***Rickettsia prowazekii*** proteins used for antigen screening and predicted characteristics according to PSORTb 3.0*. * PSORTb 3.0 is a multicomponent approach that generates likelihood scores for the localization of proteins in each of the five Gram-negative localization sites (cytoplasm, cytoplasmic membrane, periplasm, outer membrane and extracellular space). In order to generate a final prediction, the results of each module are combined and assessed; a probabilistic method and 5-fold cross validation are used to assess the likelihood of a protein being at a specific localization given the prediction of a certain module. If one of the sites has a score of 7.5 or greater, this site and its score are returned as the final prediction.
(DOCX)
