# Supplementary figures and images for: Discovery of a Protective Rickettsia prowazekii Antigen Recognized by CD8+ T Cells, RP884, Using an In Vivo Screening Platform
Source: PLoS One. 2013 Oct 16;8(10):e76253. doi: 10.1371/journal.pone.0076253 (PMC3797808; doi:10.1371/journal.pone.0076253)

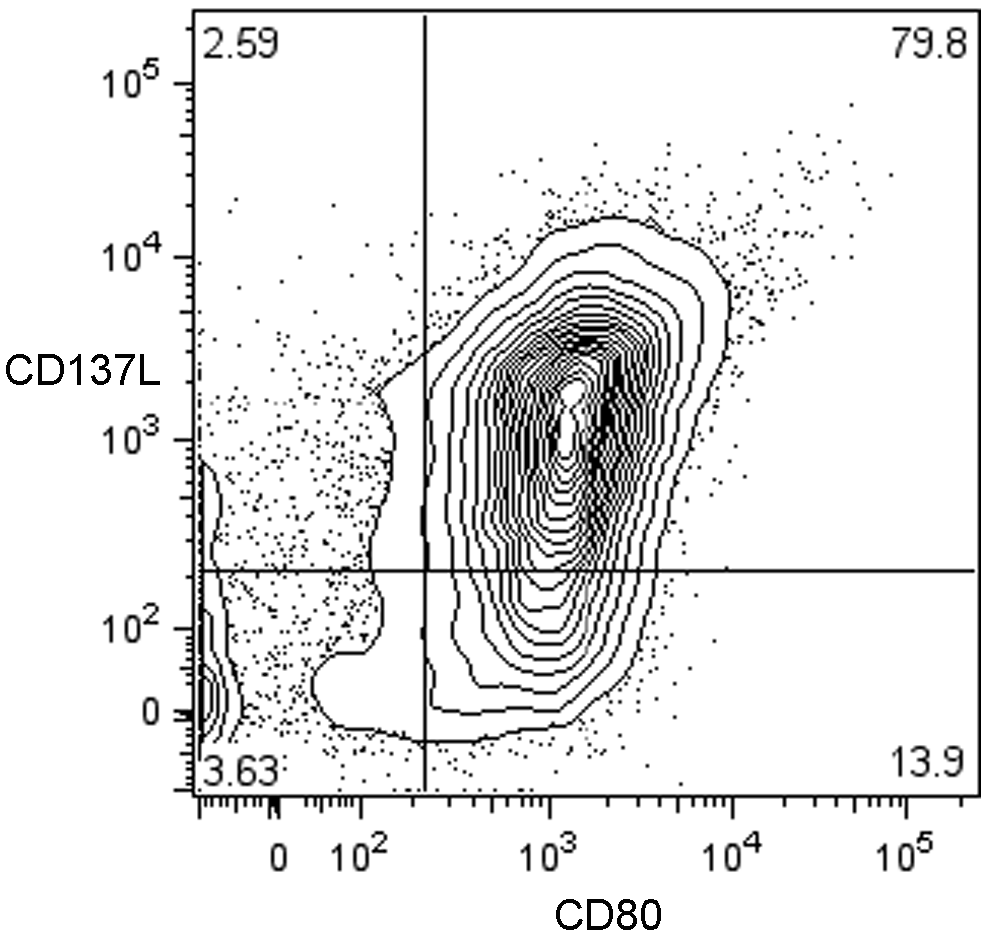

Supplement: Figure S1 — Selection and modification of antigen presenting cells (APCs). SVEC4-10 cells transduced with lentiviruses to express CD137L and CD80 were selected with puromycin and subsequently sorted by fluorescence-activated cell sorting (FACS) to isolate only cells expressing high levels of these proteins. Selected cells were expanded and analyzed by flow cytometry before nucleofection. A representative example of expanded transduced SVEC4-10 cells is shown. (TIF) [file pone.0076253.s001.tif]

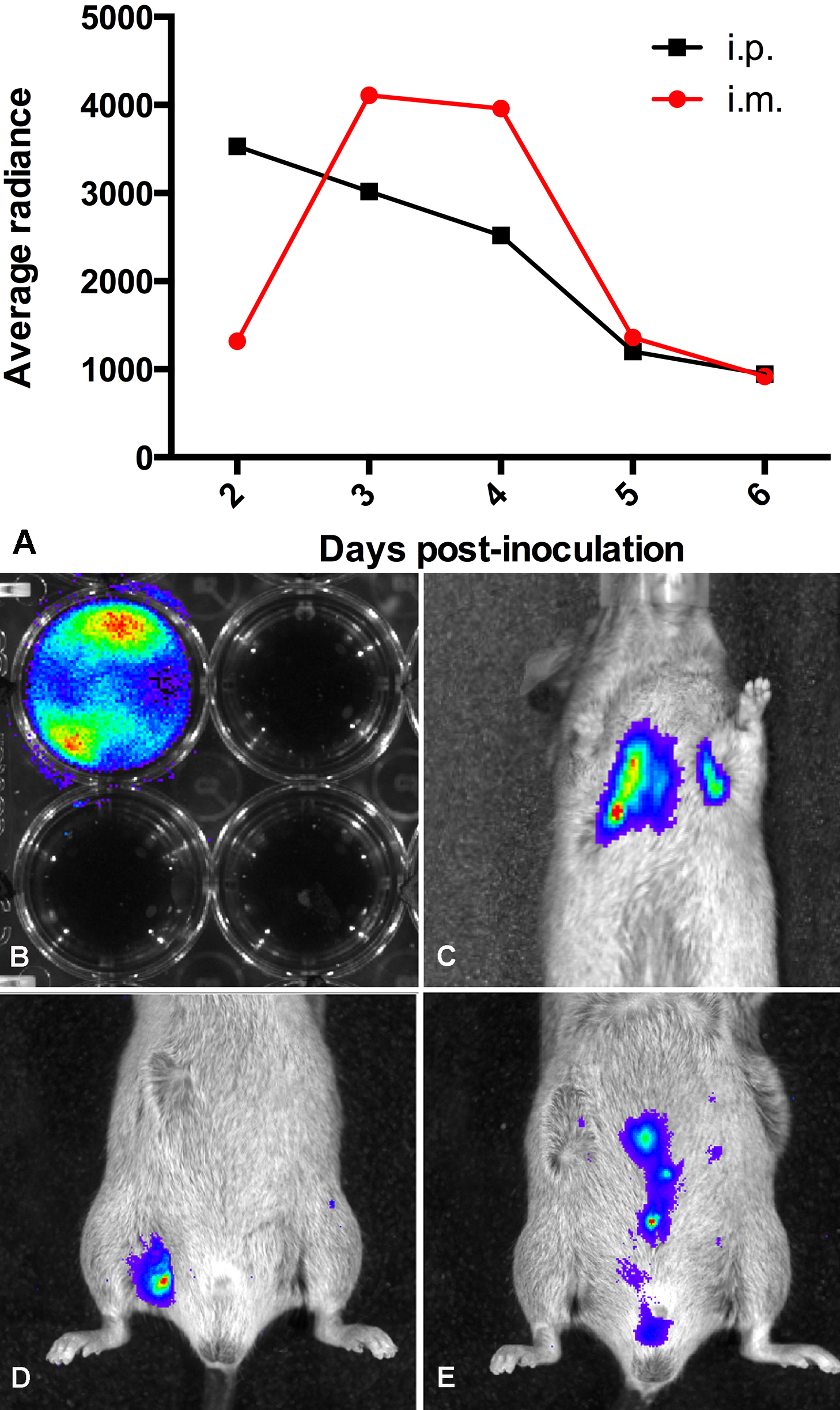

Supplement: Figure S2 — In vivo imaging of antigen presenting cells (SVEC4-10 cells) expressing luciferase at the indicated times after intraperitoneal or intramuscular injection. A) Average radiance. B) Luminescence of luciferase-expressing cells in the presence of luciferin (upper left well) vs. control cells (other wells). C) Luminescent signal from a mouse inoculated intravenously with 4×10^5^ cells expressing luciferase one hour after inoculation (average radiance is not shown in A because no luminescence was observed after one day). D) Luminescent signal from a mouse inoculated intramuscularly (i.m.) two days earlier with 4×10^5^ cells expressing luciferase. E) Luminescent signal from a mouse inoculated intraperitoneally (i.p.) two days earlier with 4 x 10^5^ cells expressing luciferase. (TIF) [file pone.0076253.s002.tif]

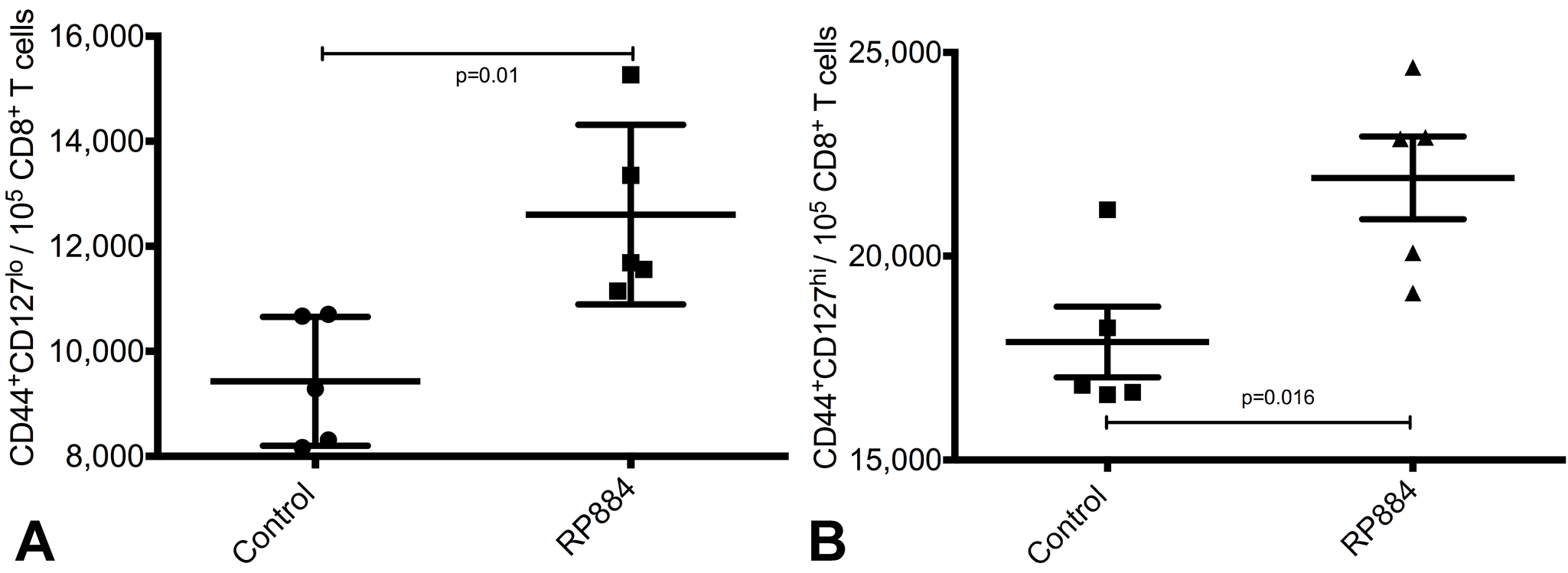

Supplement: Figure S3 — Increased effector and memory CD8+ T-cells in mice immunized with RP884. RP884-immune animals and mice immunized with the A. thaliana control gene were challenged with 6× LD50 of R. typhi and sacrificed 7 days later to obtain splenocytes for flow cytometric analysis; cells were stained with antibodies against CD3, CD8, CD44, and CD127 to determine the proportion of antigen-experienced CD8+ T-cells with an effector (panel A) or memory (panel B) phenotype based on the expression of CD127. We show individual data points, mean, and standard error of the mean (SEM). (TIF) [file pone.0076253.s003.tif]

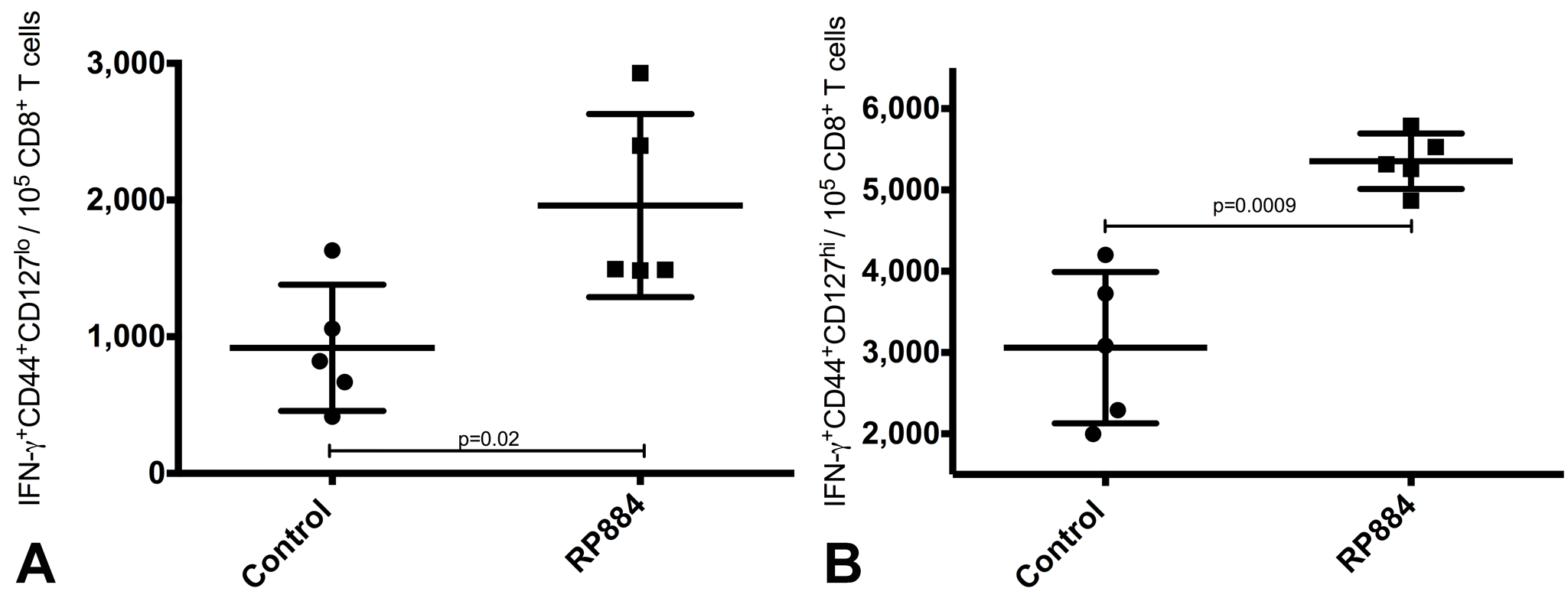

Supplement: Figure S4 — Increased IFN-γ-producing effector and memory CD8+ T-cells in mice immunized with RP884. RP884-immune animals and mice immunized with the A. thaliana control gene were challenged with 6× LD50 of R. typhi and sacrificed 7 days later (4 hours after i.p. injection of brefeldin A and monensin) to obtain splenocytes for flow cytometric analysis; cells were stained with antibodies against CD3, CD8, CD44, CD127, and IFN-γ to determine the proportion of antigen-experienced CD8+ T-cells that produce IFN-γ among effector (panel A) and memory (panel B) cells. We show individual data points, mean, and standard error of the mean (SEM). (TIF) [file pone.0076253.s004.tif]
