# Supplementary material for: Discovery of a Protective Rickettsia prowazekii Antigen Recognized by CD8+ T Cells, RP884, Using an In Vivo Screening Platform
Source: PLoS One. 2013 Oct 16;8(10):e76253. doi: 10.1371/journal.pone.0076253 (PMC3797808; doi:10.1371/journal.pone.0076253)
Supplement: File S1 — Supplementary Materials and Methods. (DOCX) [file pone.0076253.s005.docx]

Supplementary Materials and Methods:

## Generation of APC cell line expressing CD80 and CD137L

cDNA clones of mouse CD80 and mouse CD137L were obtained from Invivogen. Using AsiSI and NheI restriction enzymes, CD80 and CD137L cDNAs were removed from pORF donor vectors and cloned into pLVX-IRES-Puro (Lenti-X Lentiviral Expression System from Clontech Laboratories). This lentiviral vector was first linearized with XhoI and XbaI and an adaptor phosphorylated oligonucleotide was used to ligate the XhoI site to the AsiSI site; the XbaI site was already compatible with the NheI *s*ite. After sequencing confirmation, high-purity plasmid DNA was isolated with the QIAGEN HiSpeed Plasmid Midi Kit. For production of lentiviral particles, Lenti-X 293T Cells (Clontech Laboratories) were co-transfected with plasmid DNA and Lenti-X HTX Packaging Mix (Clontech Laboratories). Lentivirus was harvested from the supernatant, filtered through a 0.45 μm filter and concentrated with a 100 kDa Amicon Ultra Centrifugal Filter (EMD Millipore). 500 μl of concentrated lentiviral suspension was immediately used for transduction of SVEC 4-10 cells using RetroNectin (Takara Bio) following the manufacturer’s protocol. SVEC 4-10 cells were first transduced with lentiviral particles containing the mouse CD137L gene. After two weeks under puromycin selection (2 μg/ml), approximately 20 X 10^6^ cells were detached with Accumax (EMD Millipore) and stained with PE anti-mouse CD137Ligand antibody (Biolegend). Cells with the highest expression of CD137L were sorted using a FACSAria cell sorter (BD Bioscience) and subsequently transduced with the mouse CD80 lentivirus. Transduced SVEC 4-10 cells were stained with PE anti-mouse CD137L and APC anti-mouse CD80 antibodies, and the one percent of cells expressing the highest levels of both markers were sorted, amplified, and frozen in liquid nitrogen until used as antigen presenting cells (APCs).

## Quantitation of rickettsiae by quantitative real-time PCR

Primers and probes were designed using Visual OMP and ThermoBLAST software (DNAsoftware). The DNA sequences of mouse L-lactate dehydrogenase A-like 6B (*ldhal6b*) gene and rickettsial citrate synthase (*glt-A*) genes were obtained from GenBank database (http://www.ncbi.nlm.nih.gov/genbank/).

For rickettsial *glt-A* gene quantification, we amplified a 97-bp fragment using forward primer gltA-F (5’- GCTATGGGTATACCGTCGCA-3’), reverse primer gltA-R (5’- CAGGATCTTCGTGCATTTCTTTCC-3’), and TaqMan MGB (minor groove binder) glt-A-probe labeled with 5’-FAM (5’FAM- GCCATCCAACCTACGGTTCTTGC-3’). For quantification of the mouse L-lactate dehydrogenase A-like 6B (*ldhal6b*) gene, we amplified a 102-bp fragment using forward primer M-ldhal6b-F (5’-TCGGGCAGAGGCTTGGGATC-3’), reverse primer M-ldhal6b-R (5’-CGGCGATGTTCACACCACTC-3’), and TaqMan MGB M-ldhal6b-probe labeled with 5’-VIC (5’VIC-CCACCCGTGGCAGCTTTCAGAGT). Primers were ordered from Integrated DNA Technologies and probes from Applied Biosystems (Life Technologies).

Real-time PCR reactions were run using a 7900HT Fast Real-Time PCR apparatus (Applied Biosystems). The duplex real-time PCR reactions were performed with 0.25 mM of each probe, 0.35 μM of mouse-ldhal6b primers, 0.9 μM of glt-A primers, 10 μl of 2x TaqMan Gene Expression Master Mix (Applied Biosystems) and 4 μl of DNA in a final volume of 20 μl. Cycling conditions were 50°C for 2 min, 95°C for 10 min, followed by 40 cycles of 95°C for 15 seconds and 60°C for 1 minute.

## Western blot analysis

For each reaction, one million nucleofected SVEC 4-10 cells expressing single *R. prowazekii* proteins were lysed in 250 μl of freshly-made lysis buffer consisting of 1x Tris-buffered saline (TBS, pH 8.0), 100 mM Phenylmethylsulfonyl fluoride (PMSF, Sigma-Aldrich), 1% Triton X-100, and protease inhibitor cocktail (Complete mini, Roche). The protein concentration in lysates was quantified using a BCA Protein Assay kit (Pierce). 10 μg of protein in each lysate was mixed with lauryl dodecyl sulfate sample buffer (NuPAGE; Life Technologies) containing the reducing agent dithiothreitol (Life Technologies). Samples were boiled for 5 minutes prior to loading and subjected to sodium dodecyl-sulfate polyacrylamide gel electrophoresis (SDS-PAGE) in 3-N-morpholinopropanesulfonic acid (MOPS) running buffer (NuPAGE; Life Technologies) under reducing conditions in 4 to 12% gradient Bis-Tris acrylamide gel (NuPAGE; Life Technologies). A protein standard was included for molecular mass determination (Precision protein standards, broad range, prestained; Bio-Rad). Proteins were transferred to supported nitrocellulose (BA85; Schleicher & Schuell) in a semidry protein blotting unit (Bio-Rad) with 2X transfer buffer (NuPAGE; Life Technologies) at 15 V for 30 minutes.

Membranes were incubated with blocking buffer consisting of TBS (pH 8.0) with 2% nonfat milk for one hour. Primary antibodies (Living Colors DsRed Polyclonal Antibody from Clontech Laboratories) were diluted (1:1000) in the blocking buffer, and incubated overnight at 4^o^C. Membranes were washed with buffer (TBS with 0.05% Tween-20), and incubated for 1 h with Horseradish peroxidase-labeled goat anti-rabbit IgG (Promega) diluted 1:5000 in blocking buffer. Membranes were washed and SuperSignal West Pico Chemiluminescent Substrate was used to visualize bound antibody. As a loading control, membranes were stripped of bound antibody and incubated with an antibody against β-actin (Abcam) diluted 1:1000 followed by incubation with the same HRP-labeled secondary antibody.

## In vivo imaging

The Luciferase gene (*Luc2*) was a generous gift from Dr. Slobodan Paesler. The gene was PCR amplified using primers: Luc-BamHI-forw 5’-AAAGGATCCGGACCATGGAAGACGCCAAAAACATAAAG-3’ and Luc-XhoI-rev 5’- AAACTCGAGTGCACGGCGATCTTTCCGCCCTTC-3’ and cloned into pENTR 1A donor vector (Life Technologies) linearized with BamHI and XhoI. Using the Clonase LR II Enzyme Mix (Life Technologies), the Luc2 gene was transferred into our expression vector and sequence-verified. We used the same approach to nucleofect our APCs with the *Luc2*-carrying expression vector as described above in the vaccination strategy section.

Nucleofected APCs expressing the Luc2 gene were detached with Accumax (Millipor) and washed with PBS. Each mouse received 4x10^5^ cells via i.v., i.p., or intra-muscular (i.m.) route. At defined time points after APC injection, each mouse received an i.p. injection of 150 μl (15 μg/ml) of D-Luciferin (Caliper-PerkinElmer) in PBS. After 15 minutes, mice were anesthetized with Isoflurane and biophotonic imaging was performed using Perkin Elmer's IVIS-Spectrum Imaging System.

## Flow Cytometry

Spleens were collected and disaggregated in HBSS containing 2%BGS, 3mM HEPES, Golgi Plug™ and Golgi Stop™ (BD Biosciences, San Jose, CA). Mononuclear cell were purified by density gradient centrifugation (Lympholyte™-M, Cedarlane Laboratories) and stained with Live/Dead Fixable Blue (Life Technologies), APC-Alexa Fluor 750-conjugated anti-CD8 (clone 5H10), FITC-conjugated anti-CD3 (clone YCD3-1), BD Horizon V500-conjugated anti-CD44 (clone IM7), PE-Cy5 anti-CD127 (clone A7R34), PE-Cy7-conjugated anti-IFN-γ (clone XMG1.2) and Alexa Fluor 647-conjugated anti-Granzyme B (clone 16G6). CountBright™ Absolute Counting Beads (Life Technologies) were added to each sample prior to acquisition. All samples were acquired on a LSRII Fortessa cytometer (BD Biosciences); 500.000 events were captured and data were analyzed using FlowJo 9.5.3 software (Tree-Star Inc.). All analyses were performed on live CD3^+^CD8^+^ cells. Thresholds for positivity were determined with fluorescence-minus-one (FMO) control stains.

## Immunoinformatic analysis

For the computational analysis, the protein sequences from *R. prowazekii* were analyzed for the prediction of binding of 9-mer peptides to MHC class I H-2K^k^ using the following servers: NetMHCpan (http://www.cbs.dtu.dk/services/NetMHCpan/), IEBD-ANN <http://tools.immuneepitope.org/main/html/tcell_tools.html>), and SYPEITHI (http://www.syfpeithi.de/). NetMHCpan and IEBD-ANN use artificial neural networks and have been reported to outperform other servers in the prediction of known MHC class I binding epitopes and in predicting MHC binding [1-4]. Only proteins containing peptides predicted to be strong binders (IC_50_ values ≤ 50) were considered for further analysis. SYPEITHI uses the binding matrix approach for generating the S-score, which indicates how well a peptide sequence matches the canonical MHC class I binding motifs [5]. Only peptides with an S-score of 21 and higher were further analyzed. This score was arbitrarily chosen; it represents 70% of the S-score for the influenza A matrix protein epitope GILGFVFTL. We also used a predictive algorithm with a proteasome filter named RANKPEP (<http://imed.med.ucm.es/Tools/rankpep.html>). This system uses position-specific scoring matrices (PSSM) for epitope prediction, and allows performing protein sequence analysis using a proteasome filter that combines MHC class I binding affinity and proteasome processing for mouse alleles [6-8]. We used RANKPEP to analyze peptides from NetMHCpan, IEBD-AN and SYPEITHI for their likelihood to be derived from the proteasome.

Supplementary References

1 Nielsen M, Lundegaard C, Worning P, Hvid CS, Lamberth K, et al (2004) Improved prediction of MHC class I and class II epitopes using a novel Gibbs sampling approach. Bioinformatics 20: 1388-1397.

2 Nielsen M, Lundegaard C, Worning P, Lauemøller SL, Lamberth K, et al (2003) Reliable prediction of T-cell epitopes using neural networks with novel sequence representations. Protein Sci 12: 1007-1017.

3 Lin HH, Ray S, Tongchusak S, Reinherz EL, Brusic V (2008) Evaluation of MHC class I peptide binding prediction servers: applications for vaccine research. BMC Immunol 9: 8.

4 Moutaftsi M, Peters B, Pasquetto V, Tscharke DC, Sidney J, et al (2006) A consensus epitope prediction approach identifies the breadth of murine T(CD8+)-cell responses to vaccinia virus. Nat Biotechnol 24: 817-819.

5 Rammensee H, Bachmann J, Emmerich NP, Bachor OA, Stevanović S (1999) SYFPEITHI: database for MHC ligands and peptide motifs. Immunogenetics 50: 213-219.

6 Reche PA, Reinherz EL (2007) Prediction of peptide-MHC binding using profiles. Methods Mol Biol 409: 185-200.

7 Reche PA, Glutting JP, Zhang H, Reinherz EL (2004) Enhancement to the RANKPEP resource for the prediction of peptide binding to MHC molecules using profiles. Immunogenetics 56: 405-419.

8 Reche PA, Glutting JP, Reinherz EL (2002) Prediction of MHC class I binding peptides using profile motifs. Hum Immunol 63: 701-709.
