# Supplementary material for: Discovery of a Protective Rickettsia prowazekii Antigen Recognized by CD8+ T Cells, RP884, Using an In Vivo Screening Platform
Source: PLoS One. 2013 Oct 16;8(10):e76253. doi: 10.1371/journal.pone.0076253 (PMC3797808; doi:10.1371/journal.pone.0076253)
Supplement: Table S1 — Pool assignment of Rickettsia prowazekii proteins used for antigen screening and predicted characteristics according to PSORTb 3.0*. * PSORTb 3.0 is a multicomponent approach that generates likelihood scores for the localization of proteins in each of the five Gram-negative localization sites (cytoplasm, cytoplasmic membrane, periplasm, outer membrane and extracellular space). In order to generate a final prediction, the results of each module are combined and assessed; a probabilistic method and 5-fold cross validation are used to assess the likelihood of a protein being at a specific localization given the prediction of a certain module. If one of the sites has a score of 7.5 or greater, this site and its score are returned as the final prediction. (DOCX) [file pone.0076253.s006.docx]

| Rickettsial protein | Annotated function | Subcellular localization prediction | PSORTb score | Pool number |
| --- | --- | --- | --- | --- |
| RP042 | cell cycle protein MESJ (mesJ) | Cytoplasmic | 9.97 | 1 |
| RP058 | SOJ protein (soj) | Cytoplasmic | 8.96 | 1 |
| RP189 | DNA polymerase III subunit delta | Cytoplasmic | 8.96 | 1 |
| RP199 | adrenodoxin | Cytoplasmic | 8.96 | 1 |
| RP221 | threonyl-tRNA synthetase | Cytoplasmic | 10.00 | 1 |
| RP410 | UDP-N-acetylmuramoyl-L-alanyl-D-glutamate synthetase | Cytoplasmic | 9.97 | 2 |
| RP531 | translation initiation factor IF-3 | Cytoplasmic | 10.00 | 2 |
| RP585 | preprotein translocase subunit YajC | Cytoplasmic Membrane | 9.82 | 2 |
| RP688 | hypothetical protein | Cytoplasmic | 8.96 | 2 |
| RP718 | lipid A biosynthesis lauroyl acyltransferase | Cytoplasmic | 8.96 | 2 |
| RP045 | hypothetical protein | Cytoplasmic | 8.96 | 3 |
| RP168 | 16S ribosomal RNA methyltransferase RsmE | Cytoplasmic | 8.96 | 3 |
| RP192 | hypothetical protein | Cytoplasmic Membrane | 9.82 | 3 |
| RP226 | hypothetical protein | Unknown | - | 3 |
| RP875 | hypothetical protein | Cytoplasmic Membrane | 9.82 | 3 |
| RP227 | DNA topoisomerase IV subunit B | Cytoplasmic | 9.97 | 4 |
| RP336 | hypothetical protein | Cytoplasmic | 8.96 | 4 |
| RP403 | hypothetical protein | Cytoplasmic | 8.96 | 4 |
| RP485 | scaffold protein | Cytoplasmic | 9.26 | 4 |
| RP511 | hypothetical protein | Cytoplasmic | 8.96 | 4 |
| RP482 | hypothetical protein | Cytoplasmic | 9.97 | 5 |
| RP530 | branched-chain alpha-keto acid dehydrogenase subunit E2 | Cytoplasmic | 9.97 | 5 |
| RP548m | DNA repair protein RecO | Cytoplasmic | 8.96 | 5 |
| RP572 | excinuclease ABC subunit C | Cytoplasmic | 9.97 | 5 |
| RP627 | co-chaperonin GroES | Cytoplasmic | 9.26 | 6 |
| RP803 | F0F1 ATP synthase subunit alpha | Cytoplasmic | 9.97 | 6 |
| RP839 | hypothetical protein | Unknown | - | 6 |
| RP848 | hypothetical protein | Cytoplasmic | 8.96 | 6 |
| RP655 | 30S ribosomal protein S19 | Cytoplasmic | 9.26 | 7 |
| RP703 | cytochrome C-type biogenesis protein CCMF (ccmF) | Cytoplasmic Membrane | 10.00 | 7 |
| RP778 | DNA polymerase III subunit alpha | Cytoplasmic | 9.97 | 7 |
| RP884 | ferrochelatase | Cytoplasmic | 9.97 | 7 |
| RP374 | protein transport protein SEC7 (sec7) | Cytoplasmic | 8.96 | 8 |
| RP498 | cell surface antigen (sca4) | Cytoplasmic | 8.96 | 8 |
